# Influence of gut microbiota on resilience and its possible mechanisms

**DOI:** 10.7150/ijbs.82362

**Published:** 2023-05-08

**Authors:** Jianhui Wang, Ting Zhou, Feng Liu, Yan Huang, Zhiyong Xiao, Yan Qian, Wenxia Zhou

**Affiliations:** 1 Beijing Institute of Pharmacology and Toxicology, Beijing 100850, China.; 2 State Key Laboratory of Toxicology and Medical Countermeasures, Beijing 100850, China.; 3 Department of Pharmacy, The Second Affiliated Hospital of Chongqing Medical University, Chongqing, 400010, China.

**Keywords:** resilience, brain circuits, blood-brain barrier, immune system, epigenetics, gut microbiota

## Abstract

Excessive stress leads to disruptions of the central nervous system. Individuals' responses to stress and trauma differ from person to person. Some may develop various neuropsychiatric disorders, such as post-traumatic stress disorder, major depression, and anxiety disorders, while others may successfully adapt to the same stressful events. These two neural phenotypes are called susceptibility and resilience. Previous studies have suggested resilience/susceptibility as a complex, non-specific systemic response involving central and peripheral systems. Emerging research of mechanisms underlying resilience is mostly focussing on the physiological adaptation of specific brain circuits, neurovascular impairment of the blood-brain barrier, the role of innate and adaptive factors of the immune system, and the dysbiosis of gut microbiota. In accordance with the microbiota-gut-brain axis hypothesis, the gut microbiome directly influences the interface between the brain and the periphery to affect neuronal function. This review explored several up-to-date studies on the role of gut microbiota implicated in stressful events-related resilience/susceptibility. We mainly focus on the changes in behavior and neuroimaging characteristics, involved brain regions and circuits, the blood-brain barrier, the immune system, and epigenetic modifications, which contribute to stress-induced resilience and susceptibility. The perspective of the gut-brain axis could help to understand the mechanisms underlying resilience and the discovery of biomarkers may lead to new research directions and therapeutic interventions for stress-induced neuropsychiatric disorders.

## Introduction

The emerging concept of resilience is relatively novel in neuroscience, while psychologists initially defined resilience as "the process of adapting well in the face of adversity, trauma, tragedy, threats, or significant sources of stress" 1. Individuals' responses to stress and trauma can differ from person to person. Individuals with low resilience might show life-long mental disorders or relatively severe acute responses to negative stressors. However, others with higher resilience may adapt successfully to the same stressful events. As such, resilience can be considered a central role in the maturation of the nervous system and a significant target for neuropsychiatric interventions 2.

Unfortunately, the mechanism of the influence of resilience and related influencing factors is still elusive. Emerging research indicated that the homeostasis of the microbiota-gut-brain axis plays a significant role in the regulation of resilience in multiple pathways. In this paper, we reviewed studies in neuroscience to have a more comprehensive understanding of resilience and the involvement of stress-induced gut microbiota alternations. Deciphering underlying molecular mechanisms by which gut microbiota contributes to stress-induced resilience and susceptibility will help to provide intriguing and novel therapeutic interventions for stress-induced neuropsychiatric disorders.

## Implication of gut microbiota in resilience

### Definition and features of resilience

**Figure 1:**
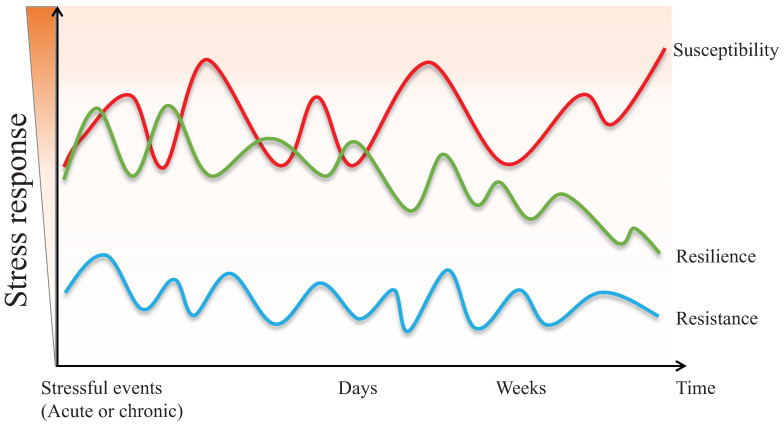
The different psychopathological responses in susceptive, resilient, and resistant individuals when exposed to acute or chronic stressful events.

Although the definition of resilience is not conclusive so far, each perspective shares three common characteristics: (1) resilience mainly describes the restoration of equilibrium or steady-state after stress stimulation. (2) resilience is not static, but a dynamic response to the environment, age, psychology, etc. (3) resilience is typically produced within serious but tolerable environmental or physiological stimuli. In other words, the process of being able to adapt well to different types of stressors is called "resilience", while the opposite one is called "susceptibility" 3, 4. Susceptibility, also referred to as vulnerability, is one in which moderate or severe levels of stress or distress persist and may increase further as more time passes 5. Furthermore, the individual, whose initial response to the stressful stimulus is absent, is defined as resistant 6. The main differences between resistant and resilient individuals lie in the initial response to stimulus and post-stress trajectory (Figure 1).

As an adapting process to adversity, resilience is a common and universal physiological phenomenon 7, 8. Research on resilience emerged in the 1970s, and most of them suggested that intrinsic psychological characteristics, including positive emotionality, were highly associated with the capacity for resilience 9, 10. In addition, different adaptive strategies may lead to resilience or susceptibility to exposure to stress. For example, an optimistic coping style promotes successful adaptation by intentionally minimizing the physical, psychological, or social damage caused by stress, while passive coping styles (such as avoidance, helplessness, etc.) can conversely lead to vulnerability and susceptibility instead of resilience 11. Given the possible impact on health, well-being, and quality of life 12, 13, a growing body of research focuses on the measurement methods and biological mechanisms of resilience.

To distinguish between resilient and susceptible individuals, most research scientists primarily measured resilience with scales. For instance, the Ego-Resiliency Scale (ERS) 14, Connor-Davidson Resilience Scale (CD-RISC) 15, Dispositional Resilience Scale (DRS) 16, and Resilience Scale 25 (RS 25) 17. However, the detailed pathways and mechanisms of resilience can be complex. With the in-depth study of the biological mechanism of resilience, the nervous system, immune system, endocrine system, genetic genes, epigenetics, and intestinal microbes, all play essential roles in the resilience process. Thereinto, stress-induced perturbed microbiota is associated with resilience, and a growing body of research has revealed a cause-effect relationship 18.

### Link between the gut microbiota and resilient behavior

Evidence is accumulating that there is a close and bidirectional symbiotic relationship between gut microbiota and the host. Notably, the impact of gut microbiota on resilience-associated behavior has been investigated. Studies revealed that the homeostasis of gut microbiota is intimately involved in the regulation of the host's psychology, emotions, and cognition in response to stressful events 19, 20.

Animal studies showed that the disordered composition of the gut microbiome contributed to susceptible behavior in rats with the learned helplessness (LH) paradigm 21. Chronic social defeat stress (CSDS) caused significant anhedonia in the mice with clear changes in microbial diversity and composition, such as *Bacteroides* spp 22, 23. Based on the arousal-based individual screening model, the resilient mice showed a decreased volatility of the gut microbiota comparing two-time points (pre-trauma and post-trauma) 24. The colonization of BALB/c and NIH Swiss mice with microbiota from each other resulted in a switch of anxiety-like phenotypes versus resilient phenotypes 25. An anhedonia-like phenotype in antibiotic-treated mice was induced by fecal microbiota transplantation (FMT) from susceptible mice 26. Moreover, germ-free mice showed increased motor activity and decreased anxiety-like behavior, accompanied by an elevated level of norepinephrine (NE), dopamine (DA), and 5-hydroxytryptamine (5-HT) in the striatum compared with control mice with normal gut microbiota 27. Another study demonstrated that germ-free mice displayed increased depression-like behaviors after the transplantation of fecal microbiota from patients with MDD 28.

Furthermore, in the human study, major depressive disorder (MDD) could exhibit the disruption of the composition of gut microbiota 29, 30. Supplementation of probiotics can also promote resilience. For example, oral treatment with *Bifidobacterium* increased the proportion of resilient mice after CSDS compared with controls 31. Treatment with *Lactobacillus rhamnosus* led to a reduction of anxiety-like behavior, alleviation of dendritic cell activation, and an increment of IL-10^+^ Tregs 32. Intakes of betaine, a dietary nutrient, have been shown to improve an anhedonia-like phenotype and decrease plasma levels of IL-6 in CSDS mice 33. Similar results were observed for the repeated administration of 3,4-Methylenedioxymethamphetamine (MDMA) 34. However, further studies are required to elucidate how the disruption of gut microbiota influences the behavior of resilience/susceptibility.

### Gut microbiota affected brain functional connectivity

The role of gut microbiota in host-brain functional connectivity has been well established 35. A growing body of research indicates the influence of the microbiota on brain structural and functional connectivity as well.

In the animal study, treating gut microbiota from attention-deficit/hyperactivity disorder persons showed a decreased functional connectivity between the right motor cortex and right visual cortex 36.

Probiotic administration was correlated with the brain activation patterns in response to emotion-related memory and task of healthy participants 37. A clinical report revealed that *Roseburia spp.*, a known butyrate producer, positively associated with the adjusted volume of the left hippocampus evaluated by magnetic resonance imaging (MRI), and also accordant negative associations between *Bacteroides sp.* (Gram-negative bacteria) and the volume of the left hippocampus 38. Additional functional MRI study demonstrated that the *Prevotella* genus (Gram-negative bacteria) was associated with the strength of all brain functional connectivity networks, while *Bifidobacterium* (Gram-positive bacteria, a species of “good bacteria”) was associated with the default mode network (DMN) and frontoparietal-attention network (FPN) 39. In patients with MDD, the fecal *Bacteroides* (Gram-negative bacteria) abundance was inversely correlated with the left dorsolateral prefrontal cortex (DLPFC) 40. Moreover, gut dysfunction also could impact the changes in neuronal integrity and edema quantified by diffusion tensor imaging (DTI) in some studies 41, 42.

### Intestinal microbiota influenced resilience-related EEG features

In recent years, electroencephalographic (EEG) signals served as an indicator of a personal capacity to cope with psychological or psychogenic stress. A study indicated higher right prefrontal theta power in resilient participants compared with susceptible participants with PTSD 43. Additionally, the EEG asymmetry across central cortical regions was found to be able to distinguish between resilience and susceptibility in children 44. The low-level resilience individual had higher alpha coherence in the right hemisphere 45. Spectral analysis of the EEG found that resilience rats had significantly greater theta power compared to susceptibility rats 46. In turn, the neurofeedback training based on the EEG alpha/theta ratio could improve resilience to stress 47.

The data from animal and human clinical studies indicated that altered intestinal microbiomes influenced EEG signals. Lipopolysaccharide intravenous injection remarkably induced excessive theta and delta activity and, these EEG abnormalities could be reversed by FMT in rats 48. Short-term administration of the probiotic of different *Lactobacillus spp.* significantly increased the ratio of EEG delta power spectra (CP2305) 49 and decreased the percentage of beta power, alpha power, and theta power in a randomized, double-blind, placebo-controlled pilot trial (PS128) 50. Gut microbiota depletion by chronic antibiotic treatment reduced the theta power density 51. In brief, the current studies have revealed the relationship between the gut microbiota and EEG features, denoting that changes to the intestinal microbiome have the potential to improve resilience-related EEG features. However, the exact metabolites that contribute to EEG regulation will be needed to ulteriorly research.

## Possible mechanisms of microbiome-mediated resilience and susceptibility

### Neural mechanisms

**Figure 2:**
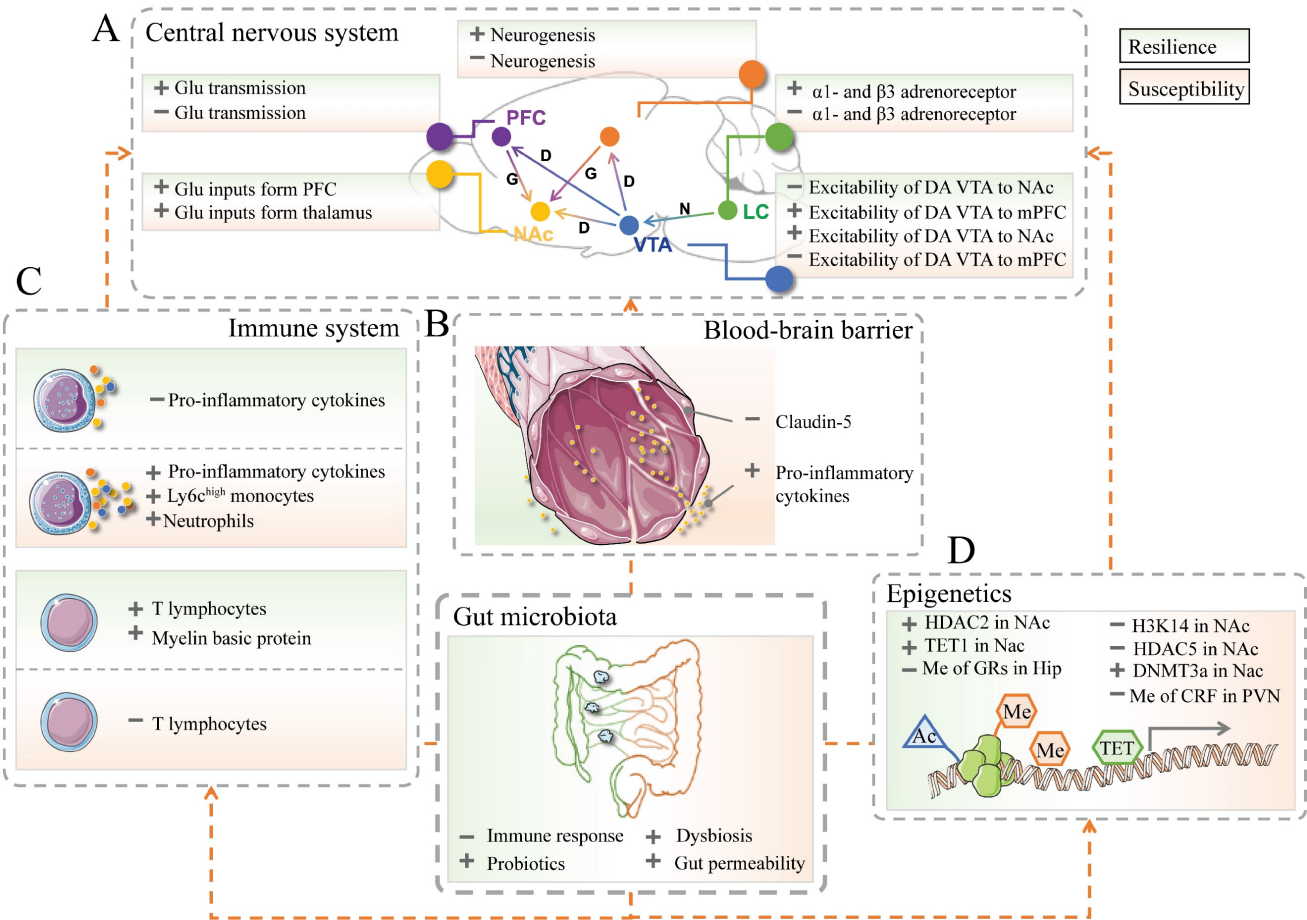
The implication of gut microbiota in resilience and susceptibility. (A) The crucial brain regions and circuits in mediating resilient and susceptible stress-induced responses. D or DA: Dopamine; G or Glu: Glutamate; HIP: Hippocampus; LC: Locus coeruleus; mPFC: Medial prefrontal cortex; N: Norepinephrine; NAc: Nucleus accumbens; PFC: Prefrontal cortex; VTA: Ventral tegmental area; light green part represents resilience; light pink part represents susceptibility. (B) The neurovascular impairments of the blood-brain barrier in stress responses. The left part in green represents resilience; the right part in pink represents susceptibility. The images were provided by Servier Medical Art 76. (C) The innate (top), and adaptive (bottom) immune systems contribute to resilience and susceptibility. (D) The epigenetic processes are associated with resilient responses. Ac: acetylation; Me: methylation; TET: ten-eleven translocation protein; HDAC: histone deacetylase; H3K14: histone 3 lysine 14; DNMTs: DNA methyltransferases; CRF: corticotropin-releasing factor. + represents up-regulation; - represents down-regulation.

Recent developments in multivariate research enable the discovery of the limbic system and its involvement in resilient and susceptible responses to stressful events. The limbic system includes the prefrontal cortex (PFC), hippocampus, amygdala, VTA-NAc reward circuit (composed of ventral tegmental area, VTA; nucleus accumbens, Nac), hypothalamus, locus coeruleus (LC), and other brain areas 52-55 (Figure 2A). For instance, the expression of brain‑derived neurotrophic factor (BDNF) pro‑peptide, IL-6, and dendritic spine density were regionally different in PFC, hippocampus and Nac, which might contribute to resilience to LH stress or CSDS 56-59.

The dopaminergic pathway in the central nervous system (CNS) is a vital reward circuit consisting of dopamine neurons, those of which located in VTA have projections throughout Nac, hippocampus, mPFC, and other prefrontal areas 60, 61. The previous literature unveiled that the gut microbiota had a role in the dopaminergic pathway. Gut microbiota composition and the microbial metabolite proved their involvement in the activity of neurotransmitters in the dopamine reward circuit and the alternation of dopamine transporter mRNA levels, which in turn are involved in social behavior deficits and psychiatric disorders 62-65. Microbiota-based interventions were proven to decrease anxiety-like behavior through the level of dopamine in the Nac and the expression of dopamine D1 and D2 receptors in the PFC 66.

Studies regarding gut microbiota proved that GABA/glutamate can be affected via the microbiota-gut-brain axis. Inhibitory GABA and excitatory glutamate are two major neurotransmitters in CNS, and they normally work together in physiological processes. Notably, imaging studies using DTI and fMRI showed that microbiota-derived fecal metabolites are correlated with functional and anatomical connectivity of the central reward network, including NAc 67. And the probiotic administration could induce GABA (B1b) mRNA alternations in a brain region-dependent manner and could prevent sCSDS-induced gene alterations that aid in signal transduction or CNS development of the NAc 68, 69. In addition, transplantation of fecal microbiota from patients with alcoholism could decrease the alpha 1 subunit of GABA type A receptor (α1GABAR) in mPFC and metabotropic glutamate receptors 1 (mGluR1) in NAc and lead to anxiety and depression in mice 70.

The hippocampus is another essential brain area that responds to stress. Numerous studies found that the administration of gut microbial metabolites or probiotics could induce alternations in the hippocampus via the microbiota-gut-brain axis, which raises the possibility that gut microbiota is effective in managing stress-related psychiatric disorders 71-73. Additionally, fecal microbiota transplantation can relieve brain injury-induced neurological deficits through the elevation of superoxide dismutase and catalase activities in the ipsilateral hippocampus 74. Gene expression in the hippocampus modified by prebiotic administration was also found to correlate with the reduction of depression- and anxiety-like behavior 75. Taken together, the emerging scientific data have supported the important role of the intestinal microbiota in the regulation of the CNS and have been highly involved in the pathogenesis of stress-related disorders. However, the precise mechanism of the microbiota in resilience-related neural circuits remained open to debate.

### Blood-brain barrier

The blood-brain barrier (BBB) consists of brain microvascular endothelial cells, astrocytes, and surrounding cells. It is a dynamic interface between peripheral circulation and CNS that selectively regulates substance exchange to contribute to the homeostasis of the CNS microenvironment 77, 78 (Figure 2B). Researchers started to realize that stress and gut microbiota could result in BBB disruption 79, 80. CSDS could induce disruption of BBB integrity via down-regulation of the endothelial tight junction protein (Claudin-5), which in turn led to the infiltration of peripheral cytokine interleukin-6 (IL-6) into the CNS and an increased proportion of stress-susceptible mice 81. LH-induced mice also showed increased permeability of the BBB. Additionally, the impaired BBB could be repaired by TNF-alpha inhibitor treatment 82. A recent study revealed that the clearance of the microbiome by antibiotics may also lead to the down-regulation of tight-junction proteins and an increase in BBB permeability. Furthermore, fecal microbiota transplantation could rescue the disruption of BBB 80. As such, gut microbiota-induced or stressful events-induced BBB impairments may be considered another therapeutic target for resilience research.

### The innate and adaptive immune system

Resilience/susceptibility of the nervous system is potentially associated with the immune system. The innate immune system recruits immune cells to infected or inflammatory areas by producing cytokines. As such, they can non-specifically recognize and defect against invading pathogens 83, 84. Animal or clinical studies suggested that pathogens or repeated psychosocial stress could influence the circulating inflammatory and induce the recruitment of immune cells (e.g., Ly6c^high^ monocytes and neutrophils) and the production of pro-inflammatory mediators (e.g., IL-1β, IL-6, and TNF-α) 85-88. The level of peripheral pro-inflammatory cytokines in patients or mice with psychiatric disorders was significantly elevated than that in healthy controls 89, 90.

Furthermore, the inhibition of IL-6 expression in CSDS mice by treatment with IL-6 antibody promotes the recovery of resilience 91, 92. In addition, lethally irradiated wild-type mice exhibited susceptibility to CSDS after transplantation of hematopoietic stem cells (HSCs) from susceptible C57BL/6J mice. On the contrary, those wild-type mice showed no susceptible phenotype to CSDS after transplanted HSCs from IL-6 knockout mice instead of those from susceptible ones 93. Chemicals that could prevent cytokine releases, such as dihydrocaffeic acid (DHCA) and malvidin-3′-O-glucoside (Mal-gluc), were proved effective in resilience development 94 (Figure 2C).

Adaptive immunity involves the later stage of infection and can be activated by exposure to invading pathogens 95. At present, there are relatively few studies on T and B lymphocytes' response when encountering stress 96. A meta-analysis of depression and immunology identified a reduction of T-cell proportion and elevation in the CD4/CD8 ratio in the blood sample of patients with MDD 97. Further studies indicated the level of T cells in CNS is positively correlated with resilience improvement in the response to psychological stress 98, 99. After receiving lymphocytes from mice exposed to CSDS, naïve lymphogenic Rag2^-/-^ mice exerted less anxious behavior and low pro-inflammatory cytokine levels compared with those transplanted with unstressed cells 100 (Figure 2C).

Interestingly, anti-inflammatory therapies may exert antidepressant effects 101. Although it is still controversial whether traditional antidepressants can decrease the level of peripheral cytokines, a recent meta-analysis showed that serum levels of interleukin-1β (IL-1β) and IL-6 were decreased after antidepressant medication treatment 102. Another study also proved that the infusion of ketamine, a rapid antidepressant, could reduce the level of pro-inflammatory cytokines 103. However, whether these inflammatory changes are causally related to the efficacy of antidepressants remains elusive.

Notably, stress-induced alternations of gut microbiota and peripheral immune system led to resilience/susceptibility development. However, the underlying mechanism is still unclear until a recent study revealed that activation of the vagus nerve by gut microbiota-mediated cytokines release could induce pro-rewarding effects in NAc, suggesting a critical role of gut microbiota in immune system-induced psychiatric disorders 104. It is well known that the α7 subtype of nicotinic acetylcholine receptors (coded by the *Chrna7* gene) and soluble epoxide hydrolase (coded by the *Ephx2* gene) played key roles in inflammation involved in MDD. FMT from *Chrna7* or *Ephx2* knock-out mice induced depression-like behaviors in antibiotic-treated mice, which were significantly blocked by the subdiaphragmatic vagotomy (SDV) 26, 105. The SDV also slowed the development of depression- and anhedonia-like phenotypes induced by ingestion of *Lactobacillus intestinalis* and *Lactobacillus reuteri* 106. These recent findings suggested that the vagus nerve was an essential part of the gut-brain axis 107.

Meanwhile, as we just mentioned above, gut microbiota or stressful events may cause BBB impairment and infiltration of cytokines into the CNS, followed by subsequent disruption of neural function and depression-like behaviors 81. Meanwhile, microglia from the mice with stressed lymphocytes shifted to an anti-inflammatory phenotype 100. Antibiotic minocycline can prevent chronic unpredictable stress-induced depressive-like behavior in rats due to inhibition of microglia activation, indicating that interventions for microglia can promote resilience 108. Since the metabolites of the gut microbiota can not only affect the immune system but also directly influence microglia 109 and BBB 110, the gut microbiota could be a novel therapeutic target for the improvement of resilience and prevent stress-related psychiatric disorders.

### Epigenetic mechanisms

Emerging evidence suggests a critical role of gut microbiota and epigenetics in resilience development. Histone modifications and DNA methylation are two major epigenetic markers implicated in stress-related disorders. Enzymes, including acetylases and methylases, are involved in those epigenetic processes (Figure 2D).

However, the role of histone acetylation in resilience is still controversial. The reduction of histone 3 lysine 14 (H3K14) acetylation in NAc was found in patients with depression and CDSD-induced susceptible mice 111. And the elevation of histone acetylation by delivery of histone deacetylase (HDACs) inhibitor in the hippocampus or NAc exerted an antidepressant effect, which is similar to that of fluoxetine 112, 113. On the contrary, another study showed the opposite effect of HDACs on resilience. CSDS-induced susceptible mice exhibited a reduction of HDAC5 in NAc instead of elevation. Additionally, chronic administration of antidepressant imipramine could significantly up-regulate HDAC5 mRNA level in the NAc, suggesting upregulation of HDAC5 in NAc may mediate a resilient response 114. Overexpression of HDAC2 in NAc by AAV-HDAC2 injection prevents social avoidance induced by chronic stress 115. Albeit the controversial results, those aforementioned findings still indicated that HDACs are highly involved in regulating resilience/susceptibility. Additionally, there is an intriguing finding that the expression of H3K14Ac in NAc is significantly different from that in other brain regions. For example, H3K14Ac levels in the hippocampus were continuously decreased after stress induction, while the levels of which in PFC were oppositely increased 116. Moreover, researchers found that HDAC inhibitors could enhance the efficacy of antidepressant treatment of the serotonin reuptake inhibitor 117. A further possibility of HDAC inhibitor treatment for clinical patients with drug-resistant depression has also been proposed 118. Taken together, HDAC may be developed as a target for the therapeutic intervention of stress-related neuropsychiatric diseases due to its involvement in resilience. Besides acetylation, histone methylation is also closely related to resilience 119.

DNA methylation refers to the process of covalent attaching methyl groups to cytosine by DNA methyltransferases (DNMTs). Methylation is an epigenetic marker for transcriptional repression through chromatin modification and alternation of DNA conformation or stability 120. Studies found that DNMT3a expression in the brain is highly associated with the separation of resilience/susceptibility phenotypes. Exposure to CSDS and subchronic variable stress (SCVS) may up-regulate the expression of DNMT3a in NAc. In addition, overexpression of DNMT3a in the NAc could result in depression-like behavior in both male and female mice, while knockout DNMAT3a in the NAc of female mice could ameliorate susceptible behavior after SCVS exposure 121, 122. The role of DNA dioxygenase, ten-eleven translocation protein 1 (TET1), in resilience has been investigated. Research detected a reduction of the level of TET1 in the NAc in CSDS -induced susceptible mice. However, the knockout of TET1 in the NAc can alleviate depression-associated behavior 123. Furthermore, an increased mRNA expression of corticotropin-releasing factor (CRF) in the paraventricular nucleus (PVN) was found in stress-induced susceptible mice, while methylation of CRF promoter was significantly reduced 124. Up-regulation of DNA methylation of glucocorticoid receptors in the hippocampus is also associated with depression-like behaviors, especially in individuals who have experienced adversity in childhood 125, 126.

Notably, the activity of those enzymes could be influenced by the host's gut microbiota and its metabolites. Probiotic intervention could restore the decrease of methylation in the hippocampus 127. Short-chain fatty acids (SCFAs), the main metabolites generated by gut microbiota, were highly involved in gut and brain interaction. Sodium butyrate, one of the SCFAs, could activate the autophagy pathway and inhibit cell proliferation via histone acetylation modification as an HDAC inhibitor 128, 129. A further study proved that butyrate treatment prevents LPS-induced depressive-like behaviors and activation of microglia in mice hippocampus by the acetylation of histone H3 and H4, suggesting an antidepressant effect 130. Besides butyrate, methyl donors (choline and folate)-induced global DNA methylation could successfully ameliorate adverse early life-related depressive-like behavior and metabolic disturbance in adult life 131, 132. The relationship between gut microbiota and epigenetic modification indicates their significant role in epigenetics involved in stress-related resilience.

## Outstanding issues

Mounting evidence from both clinical and animal studies presented convincing data that microbiota could influence the neurological, electrophysiological, and immune processes of stress-related resilience. However, our review deemed there two essential questions regarding resilience remained unanswered. Firstly, the measurement method or indicator for distinguishing resilient and susceptible individuals remains open to debate. Most human studies used scales to measure resilience, for instance, ERS, CD-RISC, DRS, RS 25, etc^17^. For animal studies, discrimination has been built on different aspects, including coping strategies 4, social symptoms of depression and anxiety 53, anhedonia 133, and urine scent marking (USM) 134. Additionally, the peptides Neuropeptide Y 135, orexins 136, sphingosine-1-phosphate 3 receptor 137, and glucocorticoid receptor 138, have been identified to segregate resilient and susceptible animals. Secondly, an explicit conceptual understanding of resilience, irrespective of the terminology being used, is essential to carry out research in this field forward. Thirdly, to determine whether gut microbiota is important in resilience phenotype, it should be researched under different conditions. For instance, if a gut microbiota is exhibited in disorder composition in animals resilient to social stress, is that same gut bacterial genus also significant in response to physical stress or predator odor? Is that same bacterial genus significant in both sexes? Is that same bacterial genus significant for outcomes in multiple response domains? In a nutshell, the causal role of the gut microbiome in genetics, epigenetics, neurobiology, and neuroendocrinology can help researchers promote the understanding of the relationship between gut microbiota and resilience.

## Conclusion and future work

An in-depth understanding of resilience could help researchers and clinicians to improve the therapeutic effects on stress-related disorders through individualized targeted therapeutic strategies. However, current studies in this area are still in their infancy, further research is still urgently required to investigate the potential mechanism and to discover potential therapeutic targets in both animal and clinical research. Accumulating evidence suggests gut microbiota influence the development of resilience in multiple pathways. This review recapitulated research on the role of gut microbiota in the stress-induced alternation of behavior, neuroimaging, CNS, BBB, immune system, and epigenetics.

Considerable development has been created in the understanding of the gut-brain axis in preclinical models of neurological disorders and the potential translation of these signs of progress to humans. A growing body of research has confirmed the cross-sectional differentiations in the composition of gut microbiota between defined diseases and controlled healthy populations. Numerous rodent models of neurological diseases have been made that imitate particular disease aspects. The FMTs from patients with certain neurological disorders into antibiotic-treated or GF rodents have led to changed cognitive and social behaviors, mimicking certain features of the human phenotype. To date, FMTs from healthy subjects into patients with neurological disorders have resulted in the amelioration of some symptoms, but not a consistent improvement. There is limited supporting evidence for the efficiency of therapies targeted at the microbiota to data. Hence, the causal relationship between the gut microbiome and resilience remains to be confirmed. Integrative studies of microbiology and neuroscience could lead to novel insight into stress-related MDD, post-traumatic stress disorder, and other neuropsychiatric disorders and effective cross-disciplinary therapeutic approaches.

## Abbreviations

5-HT: 5-hydroxytryptamine
BBB: blood-brain barrier
CNS: central nervous system
CRF: corticotropin-releasing factor
CSDS: chronic social defeat stress
DA: dopamine
DHCA: Dihydrocaffeic acid
DNMTs: DNA methyltransferases
H3K14: histone 3 lysine 14
HDACs: histone deacetylase
HPA: hypothalamic-pituitary-adrenal
HSCs: hematopoietic stem cells
IL: interleukin
LC: locus coeruleus
Mal-gluc: malvidin-3′-O-glucoside
MDD: major depressive disorder
MSNs: medium spiny neurons
NAc: nucleus accumbens
NE: norepinephrine
PFC: prefrontal cortex
PVN: paraventricular nucleus
SCVS: subchronic variable stress
TET1: ten-eleven translocation protein 1
vSub: ventral subiculum
VTA: ventral tegmental area
